# Combined Phacoemulsification and Intravitreal Dexamethasone Implant (Ozurdex®) in Diabetic Patients with Coexisting Cataract and Diabetic Macular Edema

**DOI:** 10.1155/2017/4896036

**Published:** 2017-08-13

**Authors:** Claudio Furino, Francesco Boscia, Alfredo Niro, Ermete Giancipoli, Maria Oliva Grassi, Giuseppe D'amico Ricci, Francesco Blasetti, Michele Reibaldi, Giovanni Alessio

**Affiliations:** ^1^Department of Medical Science, Neuroscience and Sense Organs, Eye Clinic, University of Bari, Bari, Italy; ^2^Department of Surgical, Microsurgical and Medical Sciences, Eye Clinic, University of Sassari, Sassari, Italy; ^3^Eye clinic, University of Catania, Catania, Italy

## Abstract

**Purpose:**

To investigate the effectiveness and safety of combined phacoemulsification and dexamethasone intravitreal implant in patients with cataract and diabetic macular edema.

**Methods:**

In this two-center, retrospective, single-group study, the charts of 16 consecutive patients who underwent combined phacoemulsification and intravitreal dexamethasone implant were retrospectively reviewed. These 16 patients, 7 men and 9 women, were observed at least 3 months of follow-up. Primary outcome was the change of the central retinal thickness (CRT); secondary outcome was the change of best-corrected visual acuity (BCVA). Any ocular complications were recorded.

**Results:**

Mean CRT decreased significantly from 486 ± 152.4 *μ*m at baseline to 365.5 ± 91 *μ*m at 30 days (*p* = .005), to 326 ± 80 *μ*m at 60 days (*p* = .0004), and to 362 ± 134 *μ*m at 90 days (*p* = .001). Mean BCVA was 20/105 (logMAR, 0.72 ± 0.34) at baseline and improved significantly (*p* ≤ .007) at all postsurgery time points. One case of ocular hypertension was observed and successfully managed with topical therapy. No endophthalmitis or other ocular complications were observed.

**Conclusion:**

Intravitreal slow-release dexamethasone implant combined with cataract surgery may be an effective approach on morphologic and functional outcomes for patients with cataract and diabetic macular edema for at least three months after surgery.

## 1. Introduction

Diabetes mellitus is associated with a 5-fold higher prevalence of cataract compared to the nondiabetic population [1]. Thus, cataract extraction is a frequently performed surgical procedure in patients with diabetes. Compared to nondiabetic cataract patients, this surgery is associated with a higher risk of complications in diabetic patients, including postsurgical development of cystoid macular edema (also called Irvine-Gass syndrome) or worsening of preexisting macular edema [2–4]. Diabetic macular edema (DME) is a complication of diabetic retinopathy and is the most common cause of visual loss in both proliferative and nonproliferative diabetic retinopathy. Approximately 20% of the patients with diabetic retinopathy are affected by macular edema [5].

Currently, there is no standard treatment approach for improving outcomes of cataract extraction in diabetic patients with different degrees of clinically significant macular edema. Previous papers proposed a combined approach with intravitreal injection of humanized anti-VEGF monoclonal antibodies (ranibizumab, bevacizumab) or triamcinolone acetonide and cataract surgery in patient with DME [6–12]. In a prospective, randomized clinical trial of intravitreous bevacizumab versus triamcinolone when administered at the time of cataract surgery, both groups gained vision but only triamcinolone acetonide was associated with a sustained reduction in central macular thickness after six months [13].

Dexamethasone intravitreal implant (Ozurdex®; Allergan Inc., Irvine, CA, USA) is a biodegradable implant that releases a small amount (700 *μ*g) of the glucocorticoid dexamethasone over a period of up to six months. Ozurdex is indicated for the treatment of adult patients with visual impairment due to diabetic macular edema who are pseudophakic or who are considered insufficiently responsive to or unsuitable for noncorticosteroid therapy or macular edema following either branch retinal vein occlusion or central retinal vein occlusion, or inflammation of the posterior segment of the eye presenting as noninfectious uveitis.

In a prospective controlled, randomized interventional pilot trial, the intravitreal dexamethasone implant at the beginning of phacoemulsification significantly reduced central macular thickness and increased visual acuity after a 24-week follow-up [14]. But only two papers analyzed the safety and efficacy of Ozurdex implant at the end of cataract surgery in patients with diabetic macular edema [15, 16].

So, in this study, we contribute to analyze the effectiveness of intravitreal administration of dexamethasone implant at the end of cataract surgery in diabetic patients with coexisting cataract and clinical significant macular edema in order to avoid any increase of macular edema following uncomplicated phacoemulsification and to obtain the better functional outcome.

## 2. Methods

This was a two-center, non-randomized, retrospective, single-group study of combined cataract surgery with intravitreal dexamethasone implant (Ozurdex) in consecutive diabetic patients with a diagnosis of cataract and clinically significant diabetic macular edema as defined by the Early Treatment Diabetic Retinopathy Study (ETDRS) [17].

The primary objective was to assess if intravitreal dexamethasone implant (Ozurdex) injection immediately after cataract surgery was able to reduce or stabilize central retinal thickness (CRT). The secondary objective was to assess changes of best-corrected visual acuity (BCVA) throughout the follow-up.

Safety evaluation has also been performed as regard intraocular pressure (IOP) variations throughout the follow-up period and incidence of other ocular adverse events (ocular inflammation and other complications, such as retinal detachment or endophthalmitis).

Patients who met all of the following criteria were considered for inclusion into the study: glycated haemoglobin ≤ 9%, controlled blood pressure (≤130/80 mmHg), visually significant cataract diagnosed using a slit lamp; nonproliferative diabetic retinopathy and clinically significant macular edema; tomographic features of nontractional diabetic macular edema, cystoid pattern, and retinal detachment pattern as described by Koleva-Georgieva [18], regardless of central retinal thickness; and proliferative diabetic retinopathy whose proliferative component had been previously treated with laser photocoagulation. Patients who met any of the following criteria were excluded from study entry: treatment of diabetic macular edema with intravitreal anti-VEGF in 3 months before surgery or any type of intravitreal corticosteroid in the 6 months before surgery; presence of untreated proliferative diabetic retinopathy; history of ocular hypertension or glaucoma; and presence of associated conditions, such as uveitis, retinal vein occlusion, and neovascular glaucoma, that could worsen macular edema. Patients who experienced intraoperative complications, such as posterior capsular tear or vitreous loss, were also excluded.

All patients underwent uneventful phacoemulsification in bag hydrophilic acrylic intraocular lens (IOL) implant using a 2.5 mm clear cornea tunnel and dispersive ophthalmic viscoelastic device. Ozurdex (700 *μ*g dexamethasone) was administered via intravitreal injection under topical anesthesia directly at the end of cataract surgery, in the inferotemporal quadrant. Patient data (age, gender, and medical history with regard to diabetes and diabetic retinopathy) were recorded from the patient's medical file. CRT was measured using optical coherence tomography (TOPCON 3D OCT-2000 or CIRRUS, Zeiss). BCVA was measured by using a standardized ETDRS protocol [17]. Testing was done at a standardized distance (4 m) under standardized lighting conditions. ETDRS values were converted into Snellen fraction and then in logMAR values for the purpose of statistical analysis. IOP was measured using a Goldmann tonometer. Measurements of BCVA and CRT at different time points of interest (baseline, 30, 60, and 90 days after surgery) were retrospectively reviewed. IOP measurements at baseline, 10, 20, 30, 40, 50, 60, and 90 days after surgery were reviewed. Ocular and systemic complications were recorded. A total of 20 patients underwent combined phacoemulsification and dexamethasone implant and met inclusion criteria. All functional and morphologic data at baseline and at all postbaseline time points up to 90 days after surgery were available for only16 patients. Four patients missed the follow-up at 90 days. 16 consecutive patients were included in this study. Statistical analysis was based on all patients included in the study. Baseline was defined as the day before surgery. Data processing, summaries, and analyses were performed using the statistical software package SAS version 9.1 or higher. A *t*-test was performed on the change from baseline in CRT to evaluate a reduction or stabilization of CRT and on the change from baseline in BCVA to evaluate an improvement of visual acuity. No formal sample size calculation was performed.

## 3. Results

**Figure 1:**
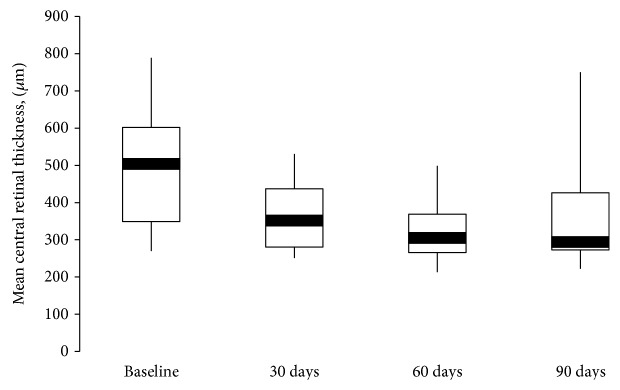
Boxplot of central retinal thickness (CRT) (*μ*m) over 90 days. Mean CRT significantly decreased (*p* ≤ .005), mainly at 60 days after combined approach.

Population's characteristics and relevant medical history data with regard to the underlying disease are summarized in Table 1. 16 consecutive patients out of 20 diabetic patients who underwent combined phacoemulsification and dexamethasone slow-release implant were included in this study. Mean age of the 7 men and 9 women included in this study was 62.5 ± 13.4 years (range: 31–76). Most patients (*n* = 15) had type 2 diabetes mellitus; only 1 patient had type 1 diabetes mellitus. The mean duration of diabetes was 20.1 ± 7.6 years and ranged between 2 and 30 years. The mean value of glycated haemoglobin (HbA1c) was 7.76 ± 0.7% (range: 6.3–9). All patients were treated for diabetes with insulin. Fourteen patients had nonproliferative diabetic retinopathy, and 2 patients with type 2 diabetes had proliferative diabetic retinopathy. The mean CRT decreased significantly from 486 ± 152.4 *μ*m at baseline to 365.5 ± 91 *μ*m at 30 days (*p* = .005), to 326 ± 80 *μ*m at 60 days (*p* = .0004), and to 362 ± 134 *μ*m at 90 days (*p* = .001) after surgery (Figure 1). The largest mean (160 ± 142 *μ*m) and median (116 *μ*m) reduction were observed at 60 days. A large standard deviation for the changes from baseline in CRT was observed (Table 2).

**Figure 2:**
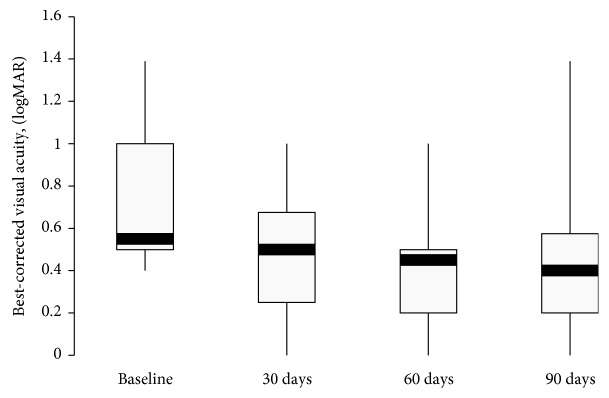
Boxplot of best-corrected visual acuity (BCVA) (logMAR) over 90 days. Mean BCVA increased significantly at all follow-up (*p* ≤ .007), mainly at 60 days after surgery.

The mean BCVA was 20/105 (logMAR, 0.72 ± 0.34) at baseline. At the postsurgery time points, the mean BCVA improved significantly to 20/60 (logMAR, 0.48 ± 0.28) at 30 days (*p* = .007), 20/53 (logMAR, 0.42 ± 0.30) at 60 days (*p* = .0008), and 20/57 (logMAR, 0.46 ± 0.39) at 90 days (*p* = .004) (Figure 2). The largest mean and median improvement of 0.30 logMAR were seen at 60 days (Table 3).

Measurements of IOP over time are summarized in Table 4. Mean and median IOP values were within normal ranges at baseline and at all postsurgery time points.

Ocular hypertension (28 mmHg) was observed in only one patient 10 days after surgery. The condition was well controlled with local therapy (dorzolamide/timolol fixed combination 2 times/day). No other ocular or systemic complications were observed.

## 4. Discussion

Phacoemulsification with in-the-bag IOL implantation, in general, do not cause progression of diabetic retinopathy [19–21]. However, previous studies suggested that diabetic patients with macular edema who were undergoing cataract surgery have poorer visual outcomes [20, 21]. So thanks to intravitreal dexamethasone implant after cataract surgery, patients with coexisting cataract and DME can benefit from downregulation of inflammatory mediators and reduction of breakdown of the blood-retinal barrier due to diabetic status and surgical inflammatory stress.

With regard to the treatment of DME, dexamethasone slow-release implant (Ozurdex) has been shown to achieve a similar rate of visual acuity improvement compared with the anti-VEGF monoclonal antibody bevacizumab, with superior anatomic outcomes and fewer injections [22].

In previous studies, we successfully experienced the use of dexamethasone implant in refractory postsurgical macular edema [23, 24]. The study reported here investigated the effect of combining an intravitreal dexamethasone implant (Ozurdex) with cataract surgery in patients with coexisting cataract and clinically significant diabetic macular edema on visual acuity, retinal thickness, and safety parameters. Sixteen patients (7 men and 9 women) with cataract and DME were included.

In a small prospective clinical trial on 18 eyes with DME, dexamethasone implant was performed at the beginning of phacoemulsification [14]. We considered performing safer implant at the end of cataract surgery when potential intraoperative complications were overcome and a better visualization of implant in the vitreous was possible. A prospective study published by Panozzo et al. [15] suggested that intravitreal dexamethasone implant performed at the end of phacoemulsification and IOL implantation was safe and effective in naïve and refractory DME. Same results were reported in a small case series which included 12 patients with macular edema secondary to diabetic retinopathy and 12 patients with macular edema secondary to retinal vein occlusion [16].

In our study, mean preoperative CRT (486 ± 152.4 *μ*m) was higher than that reported in previous papers (335.9 ± 90.6 *μ*m [14], 451 ± NR *μ*m [15], and 393 ± 166.5 *μ*m [16]) with a wide range of value (270–789 *μ*m) suggesting the heterogeneity of DME feature as a target for steroid therapy.

In our study, the mean reduction in CRT was statistically significant at 30, 60, and 90 days (*p* ≤ .005). Similar to previous reports [14–16], the greatest mean reductions in CRT occurred at 30 (120.5 *μ*m) and 60 days (160 *μ*m) after implant, with a recurrence of macular edema from the third month. After the mean reduction in CRT reaches the highest point, when dexamethasone reaches the highest concentration in the vitreous humor, the reduction in retinal thickness decreases in line with the known pharmacodynamics of the Ozurdex [25].

The mean change from baseline in BCVA was statistically significant at all follow-up visits (*p* ≤ .007) as reported by different authors [14–16]. A clinically significant improvement in BCVA has been defined as ≥0.3 logMAR [26]. Agarwal et al. [14] reported a mean visual gain of 18 letters at 12 weeks. Panozzo et al. [15] reported a stable mean visual improvement of 18 letters at month 2 after treatment. In this study, according to this cut-off value, the mean or median visual change (0.30 ± 0.28 logMAR; 0.30 logMAR) indicated a clinically significant improvement at 60 days (2 months). An improvement ≥ 0.3 logMAR at at least 1 postsurgery time point was reported in 8 patients (50%) who maintained that improvement up to 90 days. Comparing the variations in BCVA at different follow-up visits, no significant difference was observed. These results could be attributed to the weak correlation between reduction in CRT and improvement in BCVA, as reported by previous studies [27, 28]. Also, clinical characteristics such as increasing age, female sex, duration of diabetes, high HbA1c level at the time of surgery, and moderate to severe retinopathy have been associated with poor prognosis after cataract surgery in diabetic patients [29–31]. So our functional outcomes should be analyzed considering that nine patients (56.3%) were female, the mean HbA1c level before surgery was 7.76, and the mean duration of diabetes mellitus was 20.1 years.

In previous reports [14–16], the injection of Ozurdex in combination with cataract surgery raised no safety concerns with regard to IOP or other ocular or systemic complications. So, in this study, there was only one case of elevated IOP (28 mmHg) occurring 10 days after cataract extraction, which returned to normal after topic treatment. No case of endophthalmitis was observed.

The power of this study is limited by the retrospective design, small sample size, and lack of a control group. In addition, the follow-up time is too short to decide on retreatment with dexamethasone implant if macular edema should recur even if these patients were sent to injection service for the next follow-up. Another limitation was the high interpatient variability regarding the study variables CRT and BCVA at baseline, which ranged between 270 *μ*m and 789 *μ*m and between 0.09 logMAR (normal vision) and 1.00 logMAR (severe vision loss), respectively. However, that interpatient variability could suggest the efficacy of combined approach regardless of the severity of cataract and macular edema.

In conclusion, the results of this study indicate that intravitreal dexamethasone implant administration in combination with phacoemulsification and IOL implantation may be safe and effective for morphologic and visual outcomes in cataract and DME during the first 3 months after surgery.

## Figures and Tables

**Table 1:** Demographic characteristics and relevant medical history with regard to diabetes.

		Total population (*N* = 16)
Age [years]	*N*	16
	Mean (SD)	62.5 (13.4)
	Median (min, max)	67.0 (31, 76)

Gender [*n* (%)]^∗^	Male	7 (43.7%)
	Female	9 (56.3%)

Duration of diabetes [years]	Mean (SD)	20.1 (7.6)
	Median (min, max)	21.5 (2, 30)

Type of diabetes mellitus [*n* (%)]^∗^	Type 1	1 (6.3%)
	Type 2	15 (93.7%)

Treatment of diabetes [*n* (%)]^∗^	Insulin	16 (100.0%)

Hba1c [%]	Mean (SD)	7.76 (0.7)
	Median (min, max)	7.70 (6.3, 9)

Classification of diabetic retinopathy [*n* (%)]^∗^	Nonproliferative	14 (87.5%)
	Proliferative	2 (12.5%)

^∗^Percentages are based on the total number of patient.

**Table 2:** Central retinal thickness (CRT) over time.

	Measured CRT [*μ*m]			

Visit	Baseline	30 days	60 days	90 days
Mean (SD)	486 (152.4)	365.5 (90.9)	325.8 (80.4)	361.7 (133.8)
Median (min, max)	503 (270, 789)	351 (251, 531)	305 (213, 499)	294 (222, 750)

	Change from baseline in CRT [*μ*m]			

Visit	Baseline	30 days	60 days	90 days
Mean (SD)	Not applicable	−120.5 (147)	−160.0 (142)	−124 (125)
Median (min, max)		−81 (431, −41)	−116 (456, 0)	−103 (422, −18)
*p value* ^∗^		*0.005*	*0.0004*	*0.001*

^∗^Weighted *t*-test for change versus baseline.

**Table 3:** Best-corrected visual acuity (BCVA) over time.

	Measured BCVA			

Visit	Baseline	30 days	60 days	90 days
Mean (logMAR ± SD)	20/105 (0.72 ± 0.34)	20/60 (0.48 ± 0.28)	20/53 (0.42 ± 0.30)	20/57 (0.46 ± 0.39)
Median, logMAR (min, max)	0.55 (0.4, 1.39)	0.50 (0.00, 1)	0.45 (0.00, 1)	0.40 (0.00, 1.39)

	Change from Baseline in BCVA			

Visit	Baseline	30 days	60 days	90 days
Mean, logMAR ± SD	not applicable	−0.24 ± 0.30	−0.30 ± 0.28	−0.26 ± 0.30
Median, logMAR (min, max)		0.15 (−0.20, 0.99)	0.30 (−0.10, 0.99)	0.25 (−0.30, 0.99)
*p value* ^∗^		*0.007*	*0.0008*	*0.004*

^∗^Weighted t-test for change versus baseline.

**Table 4:** Intraocular pressure (IOP) over time.

	Measured IOP [mmHg]			
Visit	Baseline	10 days	20 days	30 days
Mean (SD)	15.7 (2.0)	16.0 (5.0)	14.2 (2.8)	14.5 (2.3)
Median (min, max)	15.5 (13, 20)	15.5 (9, 28)	14 (9, 18)	15 (10, 18)
Visit	40 days	50 days	60 days	90 days
Mean (SD)	15.1 (3.0)	14.9 (1.7)	14.9 (2.5)	16.4 (3.1)
Median (min, max)	15 (10, 20)	15 (11, 17)	16 (10, 18)	16.5 (11, 25)

## References

[1] Obrosova I. G., Chung S. S., Kador P. F. (2010). Diabetic cataracts: mechanisms and management. *Diabetes/Metabolism Research and Reviews*.

[2] Haddad N. M., Sun J. K., Abujaber S., Schlossman D. K., Silva P. S. (2014). Cataract surgery and in diabetic its complications patients. *Seminars in Ophthalmology*.

[3] Kim S. J., Equi R., Bressler N. M. (2007). Analysis of macular edema after cataract surgery in patients with diabetes using optical coherence tomography. *Ophthalmology*.

[4] Suto C., Hori S., Kato S. (2008). Management of type 2 diabetics requiring panretinal photocoagulation and cataract surgery. *Journal of Cataract and Refractive Surgery*.

[5] Yau J. W., Rogers S. L., Kawasaki R. (2012). Global prevalence and major risk factors of diabetic retinopathy. *Diabetes Care*.

[6] Rauen P. I., Ribeiro J. A. S., Almeida F. P. P., Scott I. U., Messias A., Jorge R. (2012). Intravitreal injection of ranibizumab during cataract surgery in patients with diabetic macular edema. *Retina*.

[7] Lanzagorta-Aresti A., Palacios-Pozo E., Menezo Rozalen J. L., Navea-Tejerina A. (2009). Prevention of vision loss after cataract surgery in diabetic macular edema with intravitreal bevacizumab: a pilot study. *Retina*.

[8] Takamura Y., Kubo E., Akagi Y. (2009). Analysis of the effect of intravitreal bevacizumab injection on diabetic macular edema after cataract surgery. *Ophthalmology*.

[9] Akinci A., Batman C., Ozkilic E., Altinsoy A. (2009). Phacoemulsification with intravitreal bevacizumab injection in diabetic patients with macular edema and cataract. *Retina*.

[10] Chen C. H., Liu Y. C., Wu P. C. (2009). The combination of intravitreal bevacizumab and phacoemulsification surgery in patients with cataract and coexisting diabetic macular edema. *Journal of Ocular Pharmacology and Therapeutics*.

[11] Habib M. S., Cannon P. S., Steel D. H. (2005). The combination of intravitreal triamcinolone and phacoemulsification surgery in patients with diabetic foveal oedema and cataract. *BMC Ophthalmology*.

[12] Akinci A., Muftuoglu O., Altınsoy A., Ozkılıc E. (2011). Phacoemulsification with intravitreal bevacizumab and triamcinolone acetonide injection in diabetic patients with clinically significant macular edema and cataract. *Retina*.

[13] Lim L. L., Morrison J. L., Constantinou M. (2016). Diabetic Macular Edema at the time of Cataract Surgery trial: a prospective, randomized clinical trial of intravitreous bevacizumab versus triamcinolone in patients with diabetic macular oedema at the time of cataract surgery - preliminary 6 month results. *Clinical and Experimental Ophthalmology*.

[14] Agarwal A., Gupta V., Ram J., Gupta A. (2013). Dexamethasone intravitreal implant during phacoemulsification. *Ophthalmology*.

[15] Panozzo G. A., Gusson E., Panozzo G., Dalla M. G. (2017). Dexamethasone intravitreal implant at the time of cataract surgery in eyes with diabetic macular edema. *European Journal of Ophthalmology*.

[16] Sze A. M., Luk F. O., Yip T. P., Lee G. K., Chan C. K. (2015). Use of intravitreal dexamethasone implant in patients with cataract and macular edema undergoing phacoemulsification. *European Journal of Ophthalmology*.

[17] (1985). Photocoagulation for diabetic macular edema. Early Treatment Diabetic Retinopathy Study report number 1. Early Treatment Diabetic Retinopathy Study research group. *Archives of Ophthalmology*.

[18] Koleva-Georgieva D., Ola M. S. (2012). Optical coherence tomography findings in diabetic macular edema. *Diabetic Retinopathy*.

[19] Shah A. S., Chen S. H. (2010). Cataract surgery and diabetes. *Current Opinion in Ophthalmology*.

[20] Squirrell D., Bhola R., Bush J., Winder S., Talbot J. F. (2002). A prospective, case controlled study of the natural history of diabetic retinopathy and maculopathy after uncomplicated phacoemulsification cataract surgery in patients with type 2 diabetes. *The British Journal of Ophthalmology*.

[21] Somaiya M. D., Burns J. D., Mintz R., Warren R. E., Uchida T., Godley B. F. (2002). Factors affecting visual outcomes after small-incision phacoemulsification in diabetic patients. *Journal of Cataract and Refractive Surgery*.

[22] Gillies M. C., Lim L. L., Campain A. (2014). A randomized clinical trial of intravitreal bevacizumab versus intravitreal dexamethasone for diabetic macular edema: the BEVORDEX study. *Ophthalmology*.

[23] Furino C., Boscia F., Recchimurzo N., Sborgia C., Alessio G. (2014). Intravitreal dexamethasone implant for macular edema following uncomplicated phacoemulsification. *European Journal of Ophthalmology*.

[24] Furino C., Boscia F., Recchimurzo N., Sborgia C., Alessio G. (2014). Intravitreal dexamethasone implant for refractory macular edema secondary to vitrectomy for macular pucker. *Retina*.

[25] Chang-Lin J. E., Attar M., Acheampong A. A. (2011). Pharmacokinetics and pharmacodynamics of a sustained-release dexamethasone intravitreal implant. *Investigative Ophthalmology & Visual Science*.

[26] Feltgen N., Neubauer A., Jurklies B. (2006). Multicenter study of the European Assessment Group for Lysin in Eye (EAGLE) for the treatment of central retinal artery occlusion: design issues and implications. EAGLE study report no.1. *Graefe's Archive for Clinical and Experimental Ophthalmology*.

[27] Blumenkranz M. S., Haller J. A., Kuppermann B. D. (2010). Correlation of visual acuity and macular thickness measured by optical coherence tomography in patients with persistent macular edema. *Retina*.

[28] Diabetic Retinopathy Clinical Research Network, Browning D. J., Glassman A. R. (2007). Relationship between optical coherence tomography-measured central retinal thickness and visual acuity in diabetic macular edema. *Ophthalmology*.

[29] Bresnick G. H. (1983). Diabetic maculopathy: a critical review highlighting diffuse macular edema. *Ophthalmology*.

[30] Nelson M. L., Martidis A. (2003). Managing cystoids macular edema after cataract surgery. *Current Opinion in Ophthalmology*.

[31] Massin P., Audren F., Haouchine B. (2004). Intravitreal triamcinolone acetonide for diabetic diffuse macular edema. *Ophthalmology*.

